# Multi-exposure electric power monitoring image fusion method without ghosting based on exposure fusion framework and color dissimilarity feature

**DOI:** 10.3389/fnbot.2022.1105385

**Published:** 2023-01-10

**Authors:** Sichao Chen, Zhenfei Li, Dilong Shen, Yunzhu An, Jian Yang, Bin Lv, Guohua Zhou

**Affiliations:** ^1^Hangzhou Xinmei Complete Electric Appliance Manufacturing Co., Ltd., Hangzhou, China; ^2^School of Electrical and Electronic Engineering, Shandong University of Technology, Zibo, China

**Keywords:** ghosting artifacts, electric power monitoring, camera response model, color dissimilarity feature, pyramid, multi-exposure image fusion

## Abstract

To solve the ghosting artifacts problem in dynamic scene multi-scale exposure fusion, an improved multi-exposure fusion method has been proposed without ghosting based on the exposure fusion framework and the color dissimilarity feature of this study. This fusion method can be further applied to power system monitoring and unmanned aerial vehicle monitoring. In this study, first, an improved exposure fusion framework based on the camera response model was applied to preprocess the input image sequence. Second, the initial weight map was estimated by multiplying four weight items. In removing the ghosting weight term, an improved color dissimilarity feature was used to detect the object motion features in dynamic scenes. Finally, the improved pyramid model as adopted to retain detailed information about the poor exposure areas. Experimental results indicated that the proposed method improves the performance of images in terms of sharpness, detail processing, and ghosting artifacts removal and is superior to the five existing multi-exposure image fusion (MEF) methods in quality evaluation.

## 1. Introduction

Since the objects are constantly in motion, compared with most natural scenes, the dynamic range of the existing ordinary cameras is very narrow (Akçay et al., 2017). Therefore, the captured image cannot have all the details in the high dynamic range (HDR) scene at disposable. Dynamic range refers to the ratio between the brightness in the brightest and darkest areas of the images. To address the issue of low dynamic range (LDR) images, we used HDR imaging technology to merge LDR images of different scenes captured into HDR images (Debevec and Malik, 2008).

At present, there are two methods for HDR imaging, namely, MEF and tone mapping. The tone mapping method requires the camera response function (CRF) for correction in the HDR imaging process and also uses the tone mapping operator to convert HDR images to LDR images that can be shown on traditional LDR devices. The MEF method directly fuses images taken at different exposure levels in the same scene to generate HDR images with rich information. It makes up for the shortcomings of the tone mapping method. Exposure evaluation, CRF correction, and tone mapping operation are not required during HDR imaging. Therefore, it saves computation costs and is widely used in high-dynamic-range imaging.

In recent years, many MEF methods have been successfully developed. According to whether the objects in the input image sequence are moving or not, they were divided into two methods, namely, the static scene MEF method (Mertens et al., 2007; Heo et al., 2010; Gu et al., 2012; Zhang and Cham, 2012; Shen et al., 2014; Ma and Wang, 2015; Nejati et al., 2017; Huang et al., 2018; Lee et al., 2018; Ma et al., 2018; Wang et al., 2019; Ulucan et al., 2021; Wu et al., 2021; Hu et al., 2022) and the dynamic scene MEF method (Li and Kang, 2012; Qin et al., 2014; Liu and Wang, 2015; Vanmali et al., 2015; Fu et al., 2016; Ma et al., 2017; Zhang et al., 2017; Hayat and Imran, 2019; Li et al., 2020; Qi et al., 2020; Jiang et al., 2022; Luo et al., 2022; Yin et al., 2022). Mertens et al. (2007) proposed a technique for fusing exposure sequences into high-quality images using multi-scale resolution. It can generate natural-color images, but the edge texture details of the fusion image are largely lost. Zhang and Cham (2012) proposed a method to process static and dynamic exposure compositions using image gradient information. This method can reduce the tedious tone mapping steps but cannot deal with the ghosts caused by the movement of objects and cameras. Gu et al. (2012) proposed a MEF method using the Euclidean metric to measure intensity distance in gradient domain feature space. It can produce fused images with rich information. Shen et al. (2014) proposed an advanced exposure fusion method. The method integrates local, global, and saliency weights into the weight processing problem. Ma and Wang (2015) proposed a patch decomposition MEF method to save running time. It improves the color appearance of the fusion image based on the decomposition of the image patches into three components, namely, average intensity, signal structure, and signal strength. Later, Ma et al. combined structural similarity with patch structure. Ma et al. (2018) proposed a MEF method to increase the perceptual quality by optimizing the color structure similarity index (MEF-SSIMc). Nejati et al. (2017) first disaggregated the source input image into basic and detail levels. Second, the exposure function is adopted to handle the weight problem. Although this method improves computational efficiency, it cannot remove the ghosts of dynamic scenes. Lee et al. (2018) designed an advanced weight function. Its function is to increase the weights of the bright regions in underexposure images and the dark regions in overexposure images while suppressing the oversaturation of these regions. Huang et al. (2018) proposed the color multi-exposure image fusion method to enhance the detailed information of fusion images. The method is based on decomposing the images into three weights, including intensity adjustment, structure preservation, and contrast extraction, and fusing them separately, preserving a great deal of detailed information for the input images. Wang et al. (2019) proposed a multi-exposure image fusion method in YUV color space. Simple detail components are used to strengthen the fused image details, which can retain the brightest and darkest area details in the HDR scene. A few pieces of literature (Ulucan et al., 2021; Wu et al., 2021; Hu et al., 2022) describe the recent results of the MEF method. Ulucan et al. (2021) designed a MEF technology to obtain accurate weights of fused images. The weight map is constructed by watershed masking and linear embedding weights. Then, the weight map and the input image are fused. This method can produce fusion images with lots of details and a good color appearance. Wu et al. (2021) presented a MEF method based on the improved exposure evaluation and the dual-pyramid model. The method can be applied in the computer vision field and the medical, remote sensing, and electrical fields. Hu et al. (2022) proposed a MEF method for detail enhancement based on homomorphic filtering. In terms of weight map calculation, threshold segmentation and Gaussian curves are utilized for processing. In terms of detail enhancement, the pyramid model of homomorphic filtering is used for processing weight maps and input image sequences.

In the dynamic scene MEF process, there is an object motion phenomenon in the input image sequence. Therefore, we should consider removing ghosting caused by object motion. Heo et al. (2010) proposed a high-dynamic-range imaging (HDRI) algorithm using a global intensity transfer function to remove ghosting artifacts. Li and Kang (2012) proposed a MEF method to remove ghosting utilizing histogram equalization and color dissimilarity feature using median filtering. Qin et al. (2014) used a random walk algorithm to maintain the content of the moving objects and provide more details. Therefore, this method can process dynamic scenes and reduce the ghosting artifacts of fused images. To increase the color brightness of the fused image, Vanmali et al. (2015) proposed a weight-forced MEF method without ghosting. Mertens et al. (2007) presented an algorithm to obtain the weighted map and used the weight-forced technology to force the weight of newly detected objects to zero. Therefore, it can produce ghost-free images with good color and texture details. Li and Kang (2012) presented a multi-exposure image fusion method based on DSIFT deghosting. It was adopted to extract the local contrast of the source image and remove the ghosting artifacts in the dynamic scene using the dense SIFT descriptor. To enhance the quality of ghost-free fusion images, Ma et al. (2017) proposed a MEF method (SPD-MEF) based on structural patch decomposition. It uses the direction of signal structure in the patch vector space to detect motion consistency, which removes ghosts. Zhang et al. (2017) introduced the inter-consistency of pixel intensity similarity in input image sequences and the intra-consistency of the interrelationships between adjacent pixels. To reduce the cost of motion estimation and accelerate MEF efficiency, Hayat and Imran (2019) presented a MEF method (MEF-DSIFT) based on dense SIFT descriptors and guided filtering. The method calculates the color dissimilarity feature using histogram equalization and median filtering, which removes the ghosting phenomenon in the MEF of dynamic scenes. Recently, Qi et al. (2020) proposed a MEF method based on feature patches. This method removes ghosts in dynamic scenes by prior exposure quality and structural consistency checking, which improves the performance of ghost removal. Li et al. (2020) proposed a fast multi-scale SPD-MEF method. It can decrease halos in static scenes and ghosting in dynamic scenes.

The available MEF methods are mainly suitable for static scene fusion, but they lack robustness to dynamic scenes, which causes a poor ghost removal effect. Therefore, this study adopts the multi-exposure image fusion method of weighted term deghosting. Based on the Ying method, an improved exposure fusion framework based on the camera response model is proposed to process input image sequences. Based on the Hayat method, an improved color dissimilarity feature is proposed for dynamic scenes, which is used to remove ghosting artifacts caused by object motion. In this study, the proposed method can generate images without ghosting fusion with pleasing naturalness and sharp texture details. Overall, the main advantages of the proposed method are summarized as follows:

(1) This study proposes an improved exposure fusion framework based on the camera response model. For the first time, the input image sequences processed by the fusion framework are used as multi-exposure input source image sequences. Through the fusion framework processing, the brightness and contrast of the source image are enhanced, and vast details are retained.
(2) The initial weight map is designed. It is obtained by calculating four weight terms, namely, local contrast, exposure feature, brightness feature, and improved color dissimilarity feature, of the input image and multiplying the four weight terms together. For dynamic scenes, an improved color dissimilarity feature is proposed based on a hybrid median filter and histogram equalization, which strengthens the sharpness of the image and has a better deghosting effect.
(3) Weighted guided image filtering (WGIF) is utilized to refine the initial weight map. The improved multi-scale pyramid decomposition model is used to add the Laplacian pyramid information to the highest level of the weighted mapping pyramid to weaken halo artifacts and retain details.

The rest of the study is organized as follows: Section 2 describes in detail the proposed multi-scale fusion deghosting method. In section 3, the effectiveness of the proposed method is obtained by analyzing the experiment results. Finally, section 4 concludes this study and makes prospects for the future.

## 2. Multi-scale image fusion ghosting removal

### 2.1. Improved exposure fusion framework based on the camera response model

There are overexposure/underexposure areas in the input image sequence. The input image sequence used for direct multi-scale image fusion may affect the contrast and sharpness of the fused images. Therefore, we transform the brightness of all images in the exposure sequence and carry out a weighted fusion of images before and after brightness transform to enhance image contrast, as in Equation (1).


(1) $$\left\{ \begin{array}{l} I_{i}(x,y)=\text{M}(x,y)◦P_{i}^{c}(x,y)\text{+}(\text{1-M}(x,y))◦P_{i}^{c^{\prime }}(x,y)\\ P_{i}^{c^{\prime }}(x,y)=g(P^{c},k_{i})=\beta P^{\gamma }={\text{e}}^{b(1-k^{a})}P^{k^{a}}(x,y) \end{array}\right.$$

where *g* is the brightness transfer function, which uses the β-γ correction model. *P*_*i*_(*x*,*y*), *I* = 1, 2, 3 …; *N* is the input image; ${P_{i}}^{\prime }\left(x,y\right)$ is the image of *P*_*i*_(*x*,*y*) brightness change in the exposure sequence; and *k*_*i*_ is the exposure rate of the *i*-th image. M is the weight map of the input image of *P*_*i*_(*x*,*y*); “◦” indicates the dot product operator; *c* is the index of three-color channels; *a* = −0.3293 and *b* = 1.1258 are the parameters of the CRF; and *I*_*i*_(*x*,*y*) is the enhancement result.

For low-light images, image brightness *L*_*i*_(*x*,*y*) is obtained using the maximal value in the three color channels in Equation (2).


(2) $$\begin{array}{l} L_{i}\left(x,y\right)=\underset{c\in \left\{R,G,B\right\}}{\max}{P_{i}}^{c}\left(x,y\right) \end{array}$$

The illumination map T estimation algorithm has been extensively studied. This study adopts the morphological closure operation to calculate the initial illumination map T_*i*_ by Fu et al. (2016), as shown in Equation (3).


(3) $$\begin{array}{l} {\text{T}}_{i}\left(x,y\right)=\frac{L_{i}\left(x,y\right)\cdot Q_{i}\left(x,y\right)}{255} \end{array}$$

where *Q*_*i*_(*x*,*y*) denotes a structural element, and “⋅” denotes an end operation. The range is mapped to [0,1] downstream operations by dividing by 255. Then, weighted guided image filtering (WGIF) (Li et al., 2014) is used to optimize the initial illumination map T_*i*_(*x*,*y*), which can better remove the halo phenomenon than the existing guided image filter (GIF). The *V* level in the *HSV* color space for the input images is regarded as the guiding image in WGIF.

It should be noted that the key point of image fusion enhancement is the design of the weight map M(*x*,*y*). The weight map M(*x*,*y*) is calculated using the method proposed by Ying et al. (2017a) in Equation (4).


(4) $$\text{M(}x\text{,}y{\text{)=(T}}_{i}^{\text{op}}(x,y){)}^{\theta }$$

where θ = 0.5 is a parameter to control the enhanced intensity and $T_{i}^{op}\left(x,y\right)$ represents the optimized illumination map. Besides, we used the Ying et al. (2017a) exposure rate determination method to obtain the best exposure rate *k*. To obtain images with good sharpness, the non-linear unsharp masking algorithm (Ngo et al., 2020) proposed by Ngo et al. is used to increase the naturalness and sharpness of fused images.

**Figure 1:**
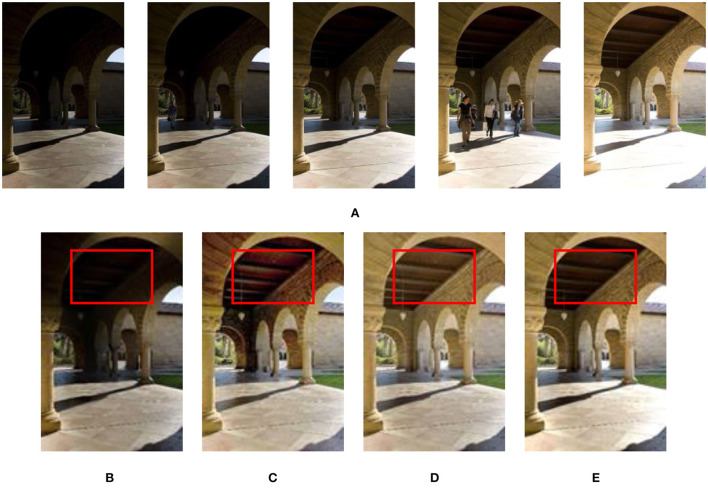
Results of dynamic scene “Arch” image sequence processed with/without CRF exposure fusion framework. **(A)** “Arch” image sequence; **(B)** Without CRF exposure fusion framework processing method (Hayat and Imran, 2019); **(C)** ICCV image processing method (Ying et al., 2017b); **(D)** CAIP image processing method (Ying et al., 2017a); **(E)** The image processing method proposed in this study.

Figure 1 shows the effect with/without CRF exposure fusion framework on experiment results. Figure 1B shows the results of the without CRF exposure fusion framework. Figures 1C–E are the result of the CRF exposure fusion framework. In Figure 1D, although the contrast of the image is improved, the image suffers from oversaturation distortion. The proposed fusion framework (see Figure 1E) significantly improves the brightness and sharpness of over/underexposure regions in the source input image sequences. Therefore, we used the proposed exposure fusion framework for related experiments in the following algorithm.

### 2.2. Multi-exposure image fusion without ghosting based on improved color dissimilarity feature and improved pyramid model

**Figure 2:**
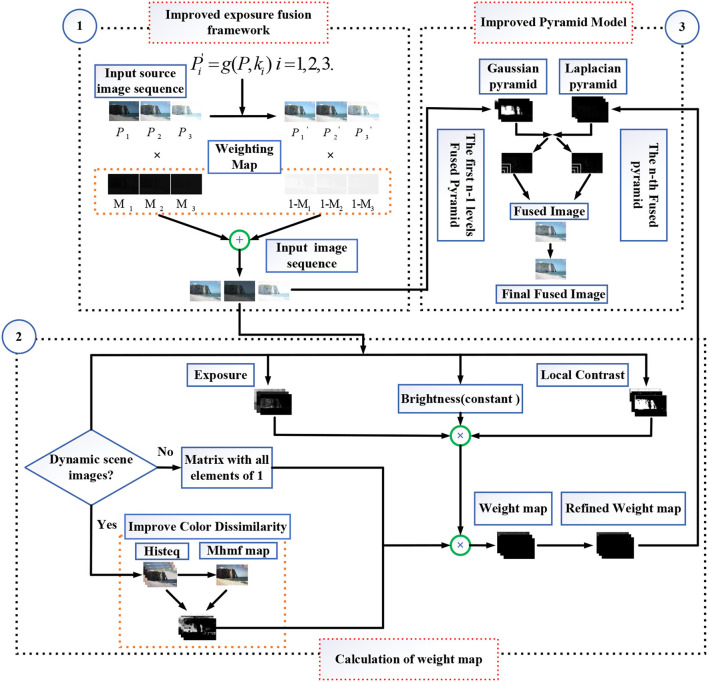
Schematic diagram of the proposed method.

This section proposes an improved multi-exposure image fusion method without ghosting. The proposed method is mainly for motion scenes in multi-exposure images. Figure 2 shows the flow schematic drawing of the proposed method.

#### 2.2.1. Improved color dissimilarity feature

An improved color dissimilarity feature based on fast multi-exposure image fusion with a median filter and recursive filter is proposed by Li and Kang (2012). Unlike the method proposed by Li and Kang (2012), static background images *I*^*S*^ of the scene are processed by a hybrid median filter (mHMF) (Kim et al., 2018) as in Equation (5).


(5) $$\begin{array}{l} I^{S}\text{=}mhmf\left(I_{\min}^{HE}\left(x,y\right)\right) \end{array}$$

where *I*^*S*^ represents the static background of the scene and *mhmf* (·) denotes an operator. The hybrid median filter (mHMF) (Kim et al., 2018) was applied to the worst image $I_{\min}^{HE}\left(x,y\right)$ in the histogram equalized exposure sequence $I_{i}^{HE}\left(x,y\right)$, which is more beneficial to preserving the image edges in regions such as mutation than the median filter. Besides, the color dissimilarity feature *D*_*i*_(*x*,*y*) of moving objects is calculated between the static background image *I*^*S*^ and histogram equalized image $I_{i}^{HE}\left(x,y\right)$ in Li and Kang (2012) and Hayat and Imran (2019).

**Figure 3:**
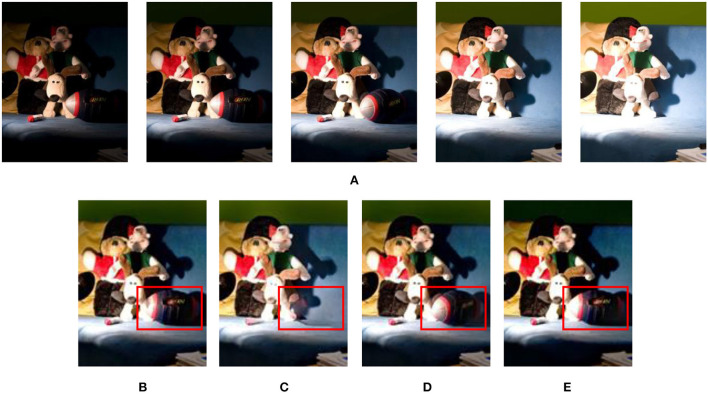
The results of processing the dynamic scene of the “Puppets” image sequence using the original/improved color dissimilarity features. **(A)** “Puppets” image sequence; **(B)** using the original color dissimilarity feature (Li and Kang, 2012); **(C)** hybrid median filter processing the brightest exposure image; **(D)** hybrid median filter processing the good exposure image; **(E)** hybrid median filter processing the darkest exposure image.

Comparisons of the color dissimilarity feature by Li and Kang (2012) and the proposed method have been conducted, as shown in Figure 3. The fused image in Figure 3B generated by the method of Li and Kang (2012) has ghosting artifacts at the ellipsoid. The proposed algorithm is validated by adopting underexposure, exposure normal, and overexposure source images, as shown in Figures 3C–E. According to Figure 3E, it can be seen that the results generated by underexposure images have a good effect on deghosting and are better than in Figure 3B. Therefore, in the following algorithm, we utilized the mHMF to handle underexposure images for related experiments.

#### 2.2.2. Exposure feature and brightness feature

Because of the correlation between the three channels in *RGB* color space, which affects the final multi-scale pyramid decomposition and fusion, the input source image is converted from *RGB* to *YUV* color space. The exposure feature weight item *E*_*i*_(*x*,*y*) of the input image is measured in the *Y* channel as in Equation (6).


(6) $$\begin{array}{l} E_{i}\left(x,y\right)=e^{-\frac{{\left[Y_{i}\left(x,y\right)-\left(1-\bar{Y_{i}}\right)\right]}^{2}}{2\sigma^{2}}} \end{array}$$

where *Y*_*i*_(*x*,*y*) is the standardized value of the *Y* channel, $Y_{i}$ is the mean value of *Y*_*i*_(*x*,*y*), and σ is a Gaussian kernel parameter taken as σ = 0.2. Besides, to increase the SNR of the input image sequence and retain the detailed information of the brightest/darkest regions, this method uses the brightness quality metric $B_{i}={Y_{i}}^{2}$ in Kou et al. (2018).

#### 2.2.3. Local contrast using dense SIFT descriptor

The local contrast is measured using Equation (7), which is extracted by non-standardized dense filtering in dense SIFT descriptor (Liu et al., 2010).


(7) $$\begin{array}{l} C_{i}\left(x,y\right)={\left||DSIFT\left(I_{i}^{\text{gray}}\left(x,y\right)\right)|\right|}_{1} \end{array}$$

where *DSIFT*(.) represents the operator that computes the non-normalized dense SIFT source image mapping, *C*_*i*_(*x*,*y*) represents a simple indicator vector for local contrast measurement, and $I_{i}^{gary}\left(x,y\right)$ denotes the grayscale image corresponding to the input image sequence *I*_*i*_(*x*,*y*). At each pixel, the $I_{i}^{gary}\left(x,y\right)$ mapping is regarded as the *l*_1_ norm of *C*_*i*_(*x*,*y*). Besides, this study selects a winner-take-all weight allocation strategy (Liu and Wang, 2015; Hayat and Imran, 2019) to obtain the final local contrast weight term $C_{i}^{final}\left(x,y\right)$.

#### 2.2.4. Estimation and refinement of the weight map

First, the following four weight items of the input image sequence are calculated: color dissimilarity feature, exposure feature, brightness feature, and local contrast. Second, weight items are multiplied to generate a weighted mapping, as in Equation (8).


(8) $$\{\begin{array}{l} W_{i}(x,y)=C_{i}^{final}(x,y)\times B_{i}\times E_{i}(x,y),\text{ for static scene}\\ W_{i}(x,y)=C_{i}^{final}(x,y)\times B_{i}\times E_{i}(x,y)\times D_{i}(x,y),\\ \text{ for dynamic scene} \end{array}$$

Using WGIF (Li et al., 2014) directly refines and filters the weight map obtained by Equation (8), which is different from the refinement of the weight map in Liu and Wang (2015) and Hayat and Imran (2019). In the process of filter refinement, both the source image and the guide image are used *W*_*i*_(*x, y*). Then, normalizing refined weight maps makes weight maps sum to 1 at every pixel. The final weight map is shown in Equation (9).


(9) $$\begin{array}{l} {\bar{W}}_{i}\left(x,y\right)={\left[\overset{N}{\underset{i=1}{\sum }}{\overset{\wedge }{W}}_{i}^{\text{WF}}\left(x,y\right)+\;\varepsilon \;\right]}^{-1}\left({\overset{\wedge }{W}}_{i}^{\text{WF}}\left(x,y\right)+\;\varepsilon \;\right) \end{array}$$

where ${Ŵ}_{i}^{WF}\left(x,y\right)$ denotes the weight map after WGIF refinement, $W_{i}\left(x,y\right)$ denotes the final normalized weight map, and ε = 10^−5^ is a small positive value, avoiding a zero denominator in the calculation process.

#### 2.2.5. Improved pyramid decomposition fusion model

**Figure 4:**
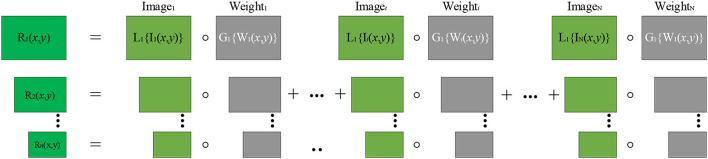
General flow of multi-scale exposure fusion. I_*i*_(*x*,*y*) is an LDR image. W_*i*_(*x*,*y*) is a weighted mapping. The Laplacian pyramid is obtained by LDR image decomposition, and the weighted mapping decomposition obtains the Gaussian pyramid. R_1_(*x*,*y*)–R_*n*_(*x*,*y*) is the resulting level of the Laplacian pyramid.

Utilizing the original multi-scale pyramid model (Mertens et al., 2007) may produce fusion images with a loss of details and the halo phenomenon. Therefore, an improved pyramid fusion model is used. In this pyramid model, the Laplacian and Gaussian pyramids are disaggregated into *n* levels, as shown in Figure 4. The total number of levels *n* is defined by Equation (10).


(10) $$\begin{array}{l} n=\left[log_{2}\left(min\left(r_{\text{o}},c_{\text{o}}\right)\right)\right]-2 \end{array}$$

where *r*_o_ and *c*_o_ are the number of rows and columns of input image pixels, respectively.

It is considered that, at the highest level of the Gaussian pyramid, improper smoothing of edges is the main reason for producing halos. On the lower levels of the Gaussian pyramid, the improper smoothing of the edges is not evident for the generation of halos. Therefore, on the *n*-th level of the RGB color space pyramid, using the single-scale fusion algorithm in Ancuti et al. (2016) adds the Laplacian pyramid information of the source image to the Gaussian pyramid weighted mapping as in Equation (11).


(11) $$\begin{array}{l} R_{n}^{i}=\left[G_{\bar{n}}\left\{{\bar{W}}_{i}\left(x,y\right)\right\}+\lambda |L_{1}\left\{I_{i}\left(x,y\right)\right\}|\right]I_{i}\left(x,y\right) \end{array}$$

where *I*_*i*_ is the input image of LDR, $R_{n}^{i}$ is the result of fusing the *i*-th image and the *i*-th image weight on the *n*-th level, and $G_{n}\left\{W_{i}\left(x,y\right)\right\}$ is the *n*-th Gaussian pyramid of $W_{i}\left(x,y\right)$. In Ancuti et al. (2016), *n* is the maximum number of levels of the Gaussian pyramid, *L*_1_{*I*_*i*_(*x, y*)} is the first level of the input image *I*_*i*_(*x*,*y*) Laplacian pyramid, and λ is the coefficient of *L*_1_{*I*_*i*_(*x, y*)}, which controls the amplitude of the high-frequency signal *L*_1_{*I*_*i*_(*x, y*) }.

To retain detailed information on overexposed/underexposed areas, on the *n*-th level, the improved multi-scale exposure fusion algorithm proposed by Wang et al. (2019) is used as in Equation (12).


(12) $$\begin{array}{l} R_{n}^{i}\left(x,y\right)=\left[G_{\bar{n}}\left\{G_{n}\left\{{\bar{W}}_{i}\left(x,y\right)\right\}\right\}+\lambda |L_{1}\left\{L_{n}\left\{I_{i}\left(x,y\right)\right\}\right\}|\right]L_{n}\left\{I_{i}\left(x,y\right)\right\} \end{array}$$

For underexposure source images, |*L*_1_{*L*_*n*_{*I*_*i*_(*x, y*)}}| in Equation (12) is introduced at the *n*-th level to correct the incorrect weights introduced by the weighted mapping smoothed by the Gaussian smoothing filter. It also reasonably enhances the weight of the well-exposure areas in the underexposure image, which retains the details of the underexposure areas. For overexposure images, the weight map of the *n*-th level adopts the primary Gaussian smoothing filter to smooth, which retains the details of the overexposure area.

For other scales, the improved pyramid fusion is the same as the original pyramid fusion (Mertens et al., 2007). Finally, reconstructing the Laplacian pyramid composed of *R*_*l*_(*x*,*y*) in Equation (13) generates the fused image *R*.


(13) $$\begin{array}{l} R_{l}\left(x,y\right)=\overset{N}{\underset{i=1}{\sum }}R_{l}^{i}\left(x,y\right),l=1,2,\ldots ,n \end{array}$$

where *l* represents the level number of the pyramid. The image details and brightness enhancement method proposed by Li et al. (2017) is adopted to enhance fusion image detail information, which obtains the final multi-scale exposure fusion image.

**Figure 5:**
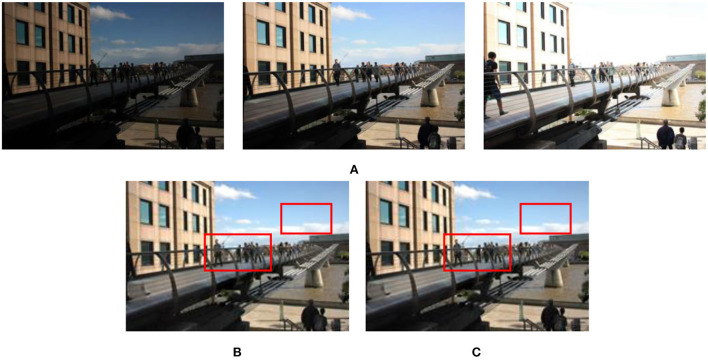
Experimental results of processing a dynamic scene “Tate” image sequence using the original/improved pyramid model. **(A)** “Tate” image sequence; **(B)** using the original pyramid model (Mertens et al., 2007); **(C)** using the improved pyramid model.

Comparisons of the original and improved pyramid models have been conducted, as shown in Figure 5. Compared with the original pyramid model (see Figure 5B), the generated image in Figure 5C by the improved pyramid model performs well in contrast and detail processing aspects, especially in pedestrian and white cloud areas. It is considered that multi-scale pyramid decomposition and fusion, loss of details, and the halo phenomenon are complex problems in pyramid decomposition and fusion. Therefore, this study selects the improved pyramid model to decompose and fuse the input image.

## 3. Experimental analysis

### 3.1. Experimental setup

**Figure 6:**
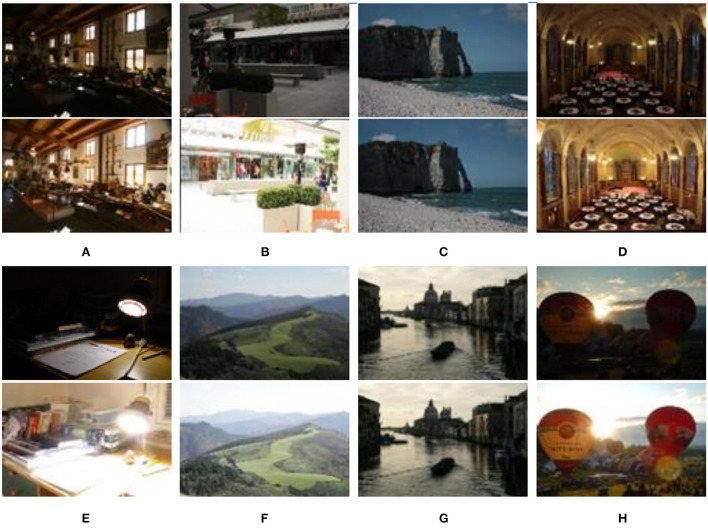
Source image sequences used in experiments. **(A)** Farmhouse; **(B)** Brunswick; **(C)** Cliff; **(D)** Llandudno; **(E)** Cadik; **(F)** Landscape; **(G)** Venice; **(H)** Balloons.

In our experiments, five and six image groups were selected from seventeen static scene (Kede, 2018) and twenty dynamic scene (DeghostingIQADatabase, 2019) image groups, respectively. As shown in Figure 6, two images with different brightnesses are extracted from the above input image sequences. We utilized eleven image groups to test five existing MEF methods and the proposed method. The five MEF methods were presented by Mertens et al. (2007), Li and Kang (2012), Liu and Wang (2015), Lee et al. (2018), and Hayat and Imran (2019), respectively. All experiments are run on MATLAB 2019a [Intel Xeon X5675 3.07 GHz desktop with 32.00 GB RAM].

### 3.2. Subjective evaluation

In this section, to thoroughly discuss the content of the experimental results, we performed a local amplification close-up shot of the results of most sequence images.

#### 3.2.1. Dynamic scenes

**Figure 7:**
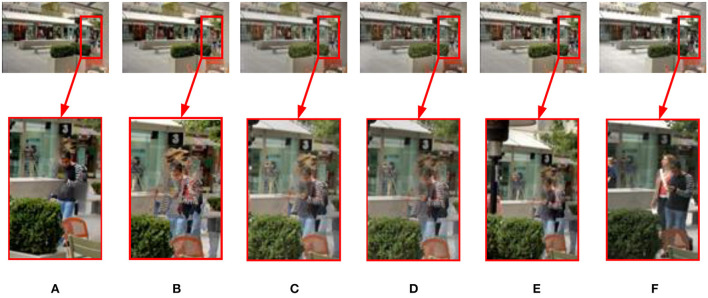
Comparison results of different methods on the dynamic “Brunswick” image sequence. **(A)** Hayat and Imran (2019); **(B)** Mertens et al. (2007); **(C)** Li and Kang (2012); **(D)** Liu and Wang (2015); **(E)** Lee et al. (2018); **(F)** the proposed method in this study.

Figure 7 shows the experimental results of different methods in the dynamic Brunswick sequence. In terms of ghost removal, the methods (see Figures 7B–E) presented by Mertens et al. (2007), Li and Kang (2012), Liu and Wang (2015), and Lee et al. (2018) have poor effects and cannot effectively remove ghosts in pedestrian areas. The pixel oversaturation distortion in Figure 7A significantly reduces the visual quality. The proposed method can produce a good result (see Figure 7F). No ghosting artifact phenomenon exists in the image, and human visual perception is natural.

**Figure 8:**
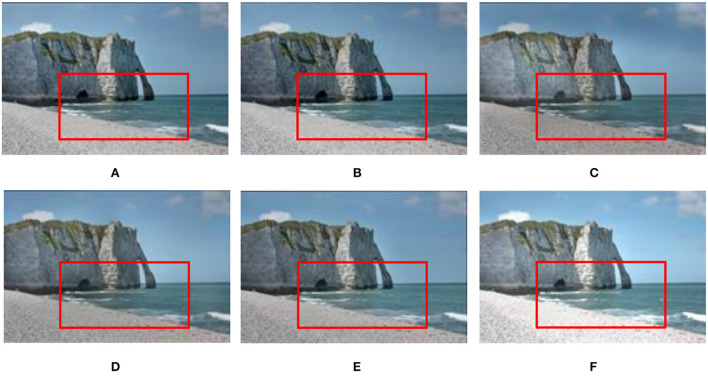
On dynamic “Cliff” image sequence, the available MEF methods compare with the proposed method. **(A)** Hayat and Imran (2019); **(B)** Mertens et al. (2007); **(C)** Li and Kang (2012); **(D)** Liu and Wang (2015); **(E)** Lee et al. (2018); **(F)** the proposed method in this study.

Figure 8 shows the fusion results of different methods in the dynamic Cliff sequence. The images in Figures 8A, B generated by the methods of Mertens et al. (2007) and Hayat and Imran (2019) are dark in color, the local contrast is not apparent, and the ghosting phenomenon exists in the water waves, which reduces the visual observation effect to a certain extent. Although the methods (see Figures 8C–E) of Li and Kang (2012), Liu and Wang (2015), and Lee et al. (2018) increase the contrast of the image, there are still darker colors and ghost phenomena. Figure 8F is the method proposed in this study. In contrast, the ghost removal performance significantly improved. On the waves and beaches, detailed information, local contrast, and naturalness are maintained, consistent with human visual observation.

**Figure 9:**
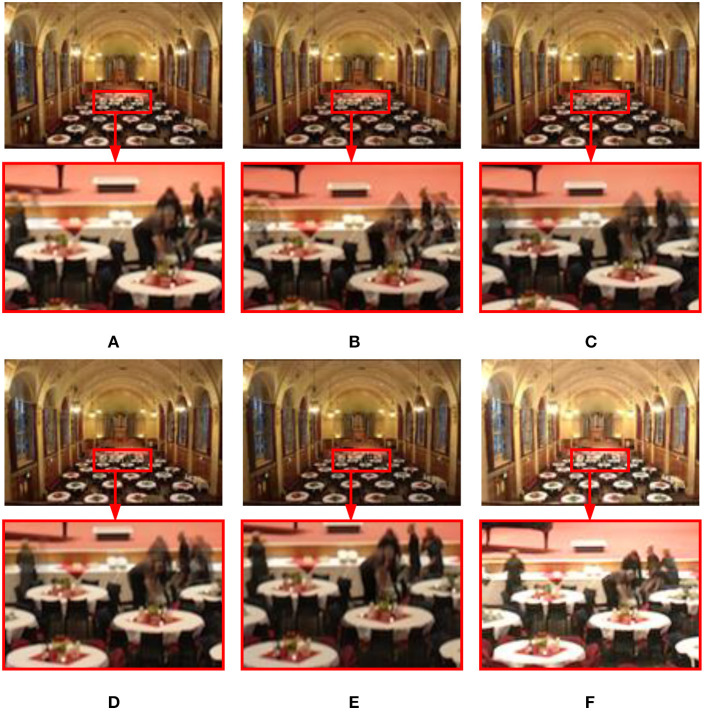
Fusion results of different methods on the dynamic “Llandudno” image sequence. **(A)** Hayat and Imran (2019); **(B)** Mertens et al. (2007); **(C)** Li and Kang (2012); **(D)** Liu and Wang (2015); **(E)** Lee et al. (2018); **(F)** the proposed method in this study.

Figure 9 shows the performance comparison of different methods in the dynamic Llandudno sequence. The results (see Figures 9B–D) acquired by Mertens et al. (2007), Li and Kang (2012), and Liu and Wang (2015) show that there are apparent ghosting artifacts in the area of characters and that there is a loss of detail information and color distortion. In Figure 9A, the overall image deghosting effect is good, but the color above the house is dark. The image in Figure 9E is unclear, and there is a color distortion phenomenon. The proposed method can produce a good result (see Figure 9F). The characters in the image have no noticeable ghosting artifacts, details are well preserved, and the exposure level is consistent with human visual observation.

#### 3.2.2. Static scenes

**Figure 10:**
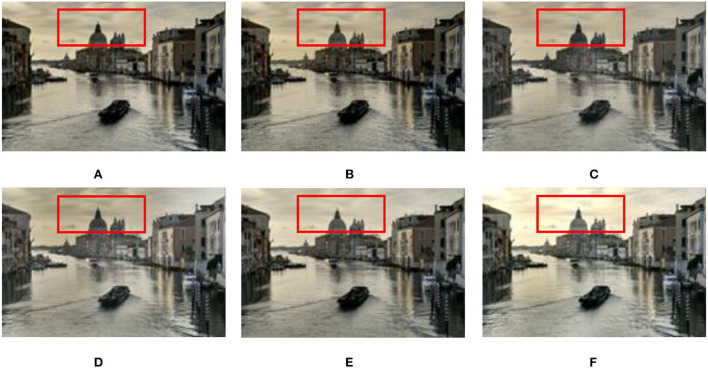
Comparison of the proposed method with Mertens et al. (2007), Li and Kang (2012), Hayat and Imran (2019), Liu and Wang (2015), and Lee et al. (2018) in the static “Venice” image sequence. **(A)** Hayat and Imran (2019); **(B)** Mertens et al. (2007); **(C)** Li and Kang (2012); **(D)** Liu and Wang (2015); **(E)** Lee et al. (2018); **(F)** the proposed method in this study.

Experimental results on the static Venice sequence using different methods are shown in Figure 10. In terms of image sharpness and detail processing, the proposed method (see Figure 10F) is superior to the methods (see Figures 10A–E) proposed by Mertens et al. (2007), Li and Kang (2012), Liu and Wang (2015), Lee et al. (2018), and Hayat and Imran (2019). Especially in Figures 10B–D, in the sky and church areas of the image, exposure and sharpness are poor, local contrast is not apparent, and fused image details are lost. In the results of the method proposed by Lee et al. (2018) and Hayat and Imran (2019), the sharpness of the fused image has improved, but there is still local contrast that is not obvious, and details are lost (see Figures 10A, E).

**Figure 11:**
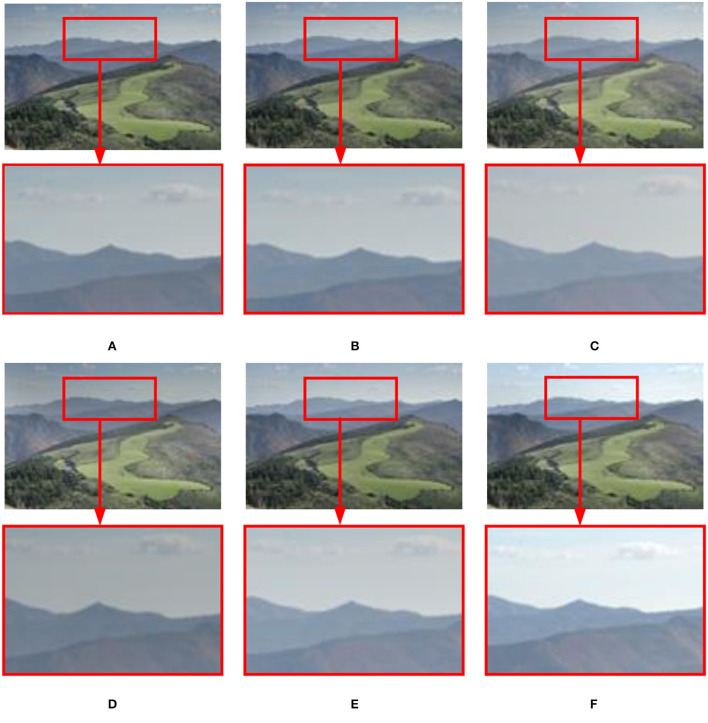
Comparison results of different methods on the static “Landscape” image sequence. **(A)** Hayat and Imran (2019); **(B)** Mertens et al. (2007); **(C)** Li and Kang (2012); **(D)** Liu and Wang (2015); **(E)** Lee et al. (2018); **(F)** the proposed method in this study.

The fusion results of six MEF methods on static scene landscape sequences are shown in Figure 11. In Figures 11B–E, in the sky area (white cloud parts), the sharpness is not good enough. In the method (see Figure 11A) proposed by Hayat and Imran (2019), although the sharpness and naturalness of the image are enhanced in the sky area, the fused image details are seriously lost. Compared with the methods (see Figures 11A–E) presented by Mertens et al. (2007), Li and Kang (2012), Liu and Wang (2015), Lee et al. (2018), and Hayat and Imran (2019), the proposed method in this study (see Figure 11F) has good saturation and contrast in the sky area, and the detailed information is retained better.

### 3.3. Objective evaluation

#### 3.3.1. Evaluation using dynamic scene structural similarity index (MEF-SSIMd)

The structural similarity index (MEF-SSIMd) (Fang et al., 2019) is applied to measure structural similarity between input image sequences and fused images in dynamic ranges. The overall MEF-SSIMd is defined in Equation (14).


(14) $$\begin{array}{l} q_{overall}=\frac{q_{s}\text{+}q_{d}}{\text{2}} \end{array}$$

where *q*_*d*_ represents MEF-SSIMd of dynamic scenes and *q*_*s*_ represents MEF-SSIMd of static scenes.

The data range of MEF-SSIMd is [0,1]. The greater the value, the better the deghosting efficiency, and the stronger the robustness of the dynamic scene. The smaller the value is, the opposite is true. As shown in Table 1, using MEF-SSIMd objectively evaluates six MEF methods for the quality of generating fused images. Overall, the proposed method is superior to the other five existing MEF methods in the performance evaluation of MEF-SSIMd.

**Table 1:** MEF-SSIMd of six MEF methods.

**Dataset**	**Hayat**	**Mertens**	**Li**	**Liu**	**Lee**	**Proposed**
Arch	**0.9503**	0.8423	0.9464	0.9417	0.8711	0.9267
Brunswick	0.8592	0.8834	0.8586	0.8261	0.8378	**0.9270**
Cliff	0.8873	0.9401	0.9243	0.9006	0.9035	**0.9687**
Llandudno	0.9072	0.8483	0.8926	0.8746	**0.9771**	0.9260
Puppets	0.8357	0.7791	0.8085	0.8035	0.8481	**0.8900**
Tate	0.8306	0.8076	0.8044	0.8298	0.8258	**0.9123**
Cadik	0.9247	0.9268	0.9032	**0.9474**	0.9290	0.9004
Landscape	0.9936	**0.9941**	0.9924	0.9883	0.9935	**0.9941**
Venice	0.8141	0.8612	0.8654	0.8250	0.8631	**0.9170**
Balloons	**0.9756**	0.9597	0.9429	0.9205	0.9539	0.9651
Farmhouse	0.9824	0.9824	0.9824	**0.9872**	0.9791	0.9588
Average	0.9055	0.8932	0.9019	0.8950	0.9075	**0.9351**
Rank	3	6	4	5	2	**1**
Total	9.9607	9.825	9.9211	9.8447	9.982	**10.2861**

The bold value indicates the maximum value, and the larger the value, the better the image fusion.

#### 3.3.2. Evaluation using natural image quality evaluator (NIQE)

In multi-exposure image fusion, the fused image should meet the requirements of the human visual system to observe the scene. Since the general purpose does not reference the IQA (image quality assessment), the algorithm requires much training to meet the IQA. Thus, a non-reference quality metric, NIQE (Mittal et al., 2012) was proposed. The smaller the NIQE value is, the better the image quality is, and the image more closely accords with the requirements of the visible human system to observe the scene. On the contrary, the greater the NIQE value is, the fewer images conform requirements of the human visual system observation scene. As shown in Table 2, NIQE is used to evaluate the quality of fusion images produced by different MEF methods. Overall, the proposed method can acquire images with better naturalness.

**Table 2:** NIQE comparison results of the MEF method.

**Dataset**	**Hayat**	**Mertens**	**Li**	**Liu**	**Lee**	**Proposed**
Arch	2.4484	2.6802	2.5456	2.6763	2.4354	2.2921
Brunswick	2.7688	**2.4740**	3.0447	3.0847	2.9462	2.8631
Cliff	3.4004	3.5940	3.4480	3.5294	3.5791	**2.9777**
Llandudno	3.1018	3.9349	3.3978	3.4781	3.9544	**2.8940**
Puppets	3.0152	**2.9780**	3.2068	3.2526	3.0909	3.2955
Tate	3.0066	**2.6109**	2.9594	2.9954	2.7553	2.8802
Cadik	3.5912	3.5379	3.7545	3.7202	3.5309	**3.4117**
Landscape	2.7917	2.8495	2.8220	2.7845	2.8128	**2.7497**
Venice	3.4862	3.8663	3.3296	3.3251	3.4182	**3.2924**
Balloons	3.3137	3.2691	3.5863	3.4309	3.4333	**3.0047**
Farmhouse	2.9762	2.9537	3.017	2.9744	2.9261	**2.7657**
Average	3.0818	3.159	3.1920	3.2047	3.1744	**2.9479**
Rank	2	3	5	6	4	**1**
Total	33.9002	34.7485	35.1117	35.2516	34.8826	32.4268

The bold value indicates the minimum value, the smaller the NIQE value is, the better the image quality is, and the image more accords with the requirements of the visible human system to observe the scene.

#### 3.3.3. Evaluation of image sharpness using local phase coherence (LPC)

In multi-exposure image fusion, sharpness is a critical factor in the visual evaluation of image quality. The sharpness of the image to achieve the human visual system can effortlessly detect blur and observe visual images. Therefore, Hassen et al. (2013) used sharpness in the complex wavelet transform domain to evaluate the local solid phase coherence (LPC) of the image features. Then, the overall sharpness index of LPC (LPC-SI) is proposed. A more considerable LPC-SI value of the fused image represents a clearer image, which conforms to the evaluation of human visual observation. A smaller LPC-SI value of the fused image represents a blurred image. The value range of LPC-SI is [0,100]. Table 3 shows the comparison results of LPC-SI values between the other five MEF methods and the presented method. A comprehensive comparison shows that the proposed method in this study outperforms the other five existing MEF methods.

**Table 3:** Test results of LPC-SI.

**Dataset**	**Hayat**	**Mertens**	**Li**	**Liu**	**Lee**	**Proposed**
Arch	0.9767	0.9710	0.9758	**0.9774**	0.9728	0.9770
Brunswick	0.9691	0.9620	0.9699	0.9703	0.9678	**0.9785**
Cliff	0.9671	0.9619	0.9637	0.9653	0.9643	**0.9777**
Llandudno	0.9737	0.9724	0.9737	0.9736	0.9734	**0.9767**
Puppets	0.9785	0.9731	0.9782	0.9763	0.9759	**0.9821**
Tate	0.9739	0.9686	0.9736	0.9723	0.9702	**0.9795**
Cadik	0.9691	0.9626	0.9655	0.9650	0.9687	**0.9700**
Landscape	0.9516	0.9484	0.9516	**0.9522**	0.9477	0.9512
Venice	0.9692	0.9633	0.9675	0.9659	0.9537	**0.9709**
Balloons	**0.9701**	0.9689	0.9696	0.9681	0.9690	0.9700
Farmhouse	0.9729	0.9728	0.9752	0.9760	0.9754	**0.9780**
Average	0.9711	0.9659	0.9695	0.9693	0.9672	**0.9738**
Rank	2	6	3	4	5	**1**
Total	10.6819	10.625	10.6643	10.6624	10.6389	**10.7116**

The bold value indicates the maximum value, a more considerable LPC-SI value of the fused image represents a clearer image, which conforms to the evaluation of human visual observation.

#### 3.3.4. Mean value analysis of objective evaluation indexes

**Figure 12:**
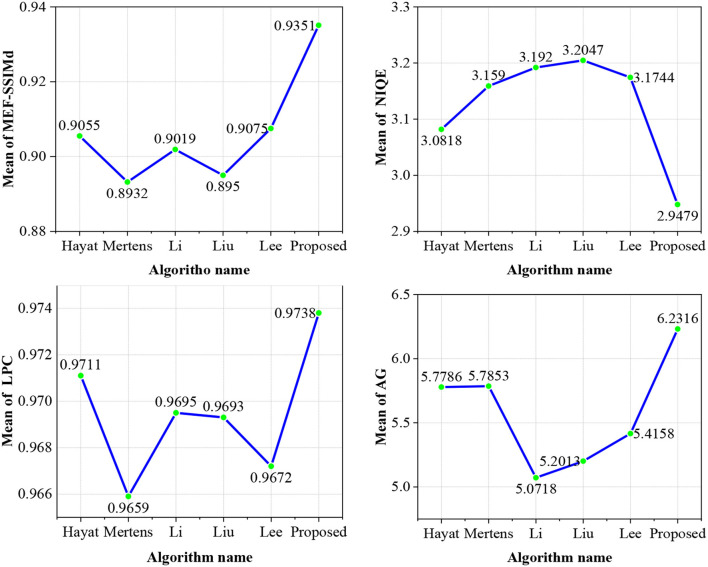
The mean values of MEF-SSIMd, NIQE, LPC-SI, and AG are obtained by different methods.

As shown in Figure 12, the proposed method in this study ranks first in the line graph of the mean values of the entire reference objective evaluation index MEF-SSIMd and non-reference objective evaluation index NIQE, LPC, and average gradient (AG). The proposed MEF method without ghosting based on the exposure fusion framework and color dissimilarity feature can effectively remove ghosting in dynamic scene MEF. It also improves the sharpness and naturalness of the fused image and retains many details.

## 4. Conclusion

An improved MEF method has been proposed in this study without ghosting based on the exposure fusion framework and color dissimilarity feature. It generates ghost-free, high-quality images with good sharpness and rich details. The proposed algorithm in this study can be further applied to power system monitoring and unmanned aerial vehicle monitoring fields. An improved exposure fusion framework based on the camera response model has been utilized to improve the contrast and sharpness of over/underexposure regions in the input image sequence. The WGIF refined weight map with an improved color dissimilarity feature was adopted to remove ghosting artifacts and to retain more image details utilizing an improved pyramid model. In the experimental tests of qualitative and quantitative evaluation for eleven image groups, including five static scene image groups and six dynamic scene image groups, this method ranks first compared with the five available MEF methods. However, when objects move frequently or move more widely, the fusion results may produce ghosting artifacts. Therefore, we hope that the researchers further study to overcome the above problems.

## Data availability statement

Publicly available datasets were analyzed in this study. This data can be found here: https://github.com/h4nwei/MEF-SSIMd.

## Author contributions

SC and ZL: conceptualization, methodology, software, and validation. DS: data curation. ZL: writing and original draft preparation. YA: writing, review, and editing. JY, BL, and SC: visualization. GZ: funding acquisition. All authors agreed to be accountable for the content of the study. All authors contributed to the article and approved the submitted version.
